# Potato Virus Y Detection in Seed Potatoes Using Deep Learning on Hyperspectral Images

**DOI:** 10.3389/fpls.2019.00209

**Published:** 2019-03-01

**Authors:** Gerrit Polder, Pieter M. Blok, Hendrik A. C. de Villiers, Jan M. van der Wolf, Jan Kamp

**Affiliations:** ^1^Agro Food Robotics, Wageningen University & Research, Wageningen, Netherlands; ^2^Biointeractions & Plant Health, Wageningen University & Research, Wageningen, Netherlands; ^3^Field Crops, Wageningen University & Research, Lelystad, Netherlands

**Keywords:** crop resistance, phenotyping, hyperspectral imaging, classification, convolutional neural network, *Solanum tuberosum*

## Abstract

Virus diseases are of high concern in the cultivation of seed potatoes. Once found in the field, virus diseased plants lead to declassification or even rejection of the seed lots resulting in a financial loss. Farmers put in a lot of effort to detect diseased plants and remove virus-diseased plants from the field. Nevertheless, dependent on the cultivar, virus diseased plants can be missed during visual observations in particular in an early stage of cultivation. Therefore, there is a need for fast and objective disease detection. Early detection of diseased plants with modern vision techniques can significantly reduce costs. Laboratory experiments in previous years showed that hyperspectral imaging clearly could distinguish healthy from virus infected potato plants. This paper reports on our first real field experiment. A new imaging setup was designed, consisting of a hyperspectral line-scan camera. Hyperspectral images were taken in the field with a line interval of 5 mm. A fully convolutional neural network was adapted for hyperspectral images and trained on two experimental rows in the field. The trained network was validated on two other rows, with different potato cultivars. For three of the four row/date combinations the precision and recall compared to conventional disease assessment exceeded 0.78 and 0.88, respectively. This proves the suitability of this method for real world disease detection.

## Introduction

In temperate regions, the main diseases of the seed potato crop are caused by viruses and bacterial infections (Dickeya and Pectobacterium). In the Netherlands, the world’s major supplier of certified seed potatoes, these two diseases are responsible for an average 14.5% declassification of seed lots (over the period 2009–2016) and an average 2.3% rejection (source: Dutch General Inspection Service NAK). This results in a total value decrease of almost 20 million euros per year for all Dutch producers.

A potato crop can be challenged by various viral pathogens resulting in a broad spectrum of different symptoms. PVY (genus Potyvirus, family Potyviridae), is one of the most prevalent and important viruses in potatoes globally (Valkonen, 2007) and is in the top–ten of most damaging plant viruses (Scholthof et al., 2011). Different strains of PVY have been identified that vary in symptom expression, including mosaic leaf discolorations caused by PVY^O^, stipple streak caused by PVY^c^, necrotic leaf spots caused by PVY^N^ and PVY^NTN^ and necrotic spots on tubers caused by PVY^NTN^ (Verma et al., 2016a,b). In the Netherlands, PVY^O^, and the recombinant strains PVY^NTN^ and PVY^N-Wi^ prevail (Verbeek et al., 2009).

There is a lack of efficient resistance in cultivated varieties, but symptom expression is variety dependent. Cultivars such as Russet Norkotah and Shepody rarely show symptoms and if so, only very mild symptoms. Nevertheless, infections of these cultivars with PVY often result in a decrease in marketable yield (Hane and Hamm, 1999). The symptomless infected plants can also be a reservoir for transmission by aphids (Draper et al., 2002).

Management of PVY is predominantly based on the use of certified, pathogen free seed, the exclusion of virus infections by roguing of symptomatic plants that can serve as inoculum source, and an early harvest, before winged aphids occur that spread the virus (Woodford, 1992; Robert et al., 2000). In addition, sanitizing tools, planters and cultivators, weed control, in particular of solanaceous species, removal of volunteer potato plants and the use of mineral oils to reduce spread of aphids, are used in management practices. Insecticides have a low effect on the transmission of the virus, as the aphids often transmit the PVY before they are killed (Shanks and Chapman, 1965; Gibson et al., 1982; Boiteau et al., 1985; Lowery and Boiteau, 1988; Boiteau and Singh, 1999; Boquel et al., 2013, 2014).

In order to prevent declassification, farmers put in a lot of effort to detect diseased plants and remove them before inspection by the Dutch General Inspection Service (NAK). The average input of manpower is estimated to be 6,2 h/ha (Kwin, 2015). Manual selection by visual observations, is labor intensive and cumbersome, in particular late in the growing season when the crop is fully developed. The cost related to plant selection by farmers is about 8 Million euros per year (40.000 ha, 6,2 h/ha, av. labor cost: €32,50/hr). In addition, the availability of skilled selection workers is getting more and more a problem. Specialized farmers that are unable to do the selection work themselves have increasing problems hiring extra selection capacity. Furthermore, human inspection is prone to FNs in which sick/diseased potato plants can be missed. This is especially the case when inspecting potato varieties with mild disease symptoms.

Visual crop inspections are done one to three times annually by staff of national inspection agencies. The reliability of their visual observations compared with a laboratory assay (PCR/ELISA) was found to be high (93%) for symptoms caused by viral diseases (K. Boons, NAK, unpublished results).

Precision agriculture together with computer vision technologies can be an alternative for human inspection (Bechar and Vigneault, 2016). High tech vision solutions can mitigate the concerns from the high labor cost and increasing potato devaluation costs. If an autonomous machine can replace a human inspector and meanwhile improve the selection quality, this might provide a new business model that is based on these high-tech solutions.

Hyperspectral sensors and imaging techniques have shown a high potential for providing new insights into plant–pathogen interactions and the detection of plant diseases (Mahlein et al., 2018). In hyperspectral imaging, every single pixel consists of an array of values, corresponding to the reflectance, emission or transmission at a certain wavelength (Behmann et al., 2015; van der Heijden and Polder, 2015). Currently, a wide variety of hyperspectral sensors are entering the market. From traditionally pushbroom line scan sensors (Polder et al., 2003) up to miniaturized handheld ‘snap shot’ cameras (Behmann et al., 2018). From a technical perspective, hyperspectral imaging has a lot of advantages compared to other visual rating and detection methods. Hyperspectral sensors are able to measure pathogen-induced changes in plant physiology non-invasively and objectively (Thomas et al., 2018).

Several studies showed that hyperspectral imaging is an especially valuable tool for disease detection in a range of different crops on different scales from the tissue to the canopy level (Sankaran et al., 2010; Mahlein et al., 2012; Wahabzada et al., 2015; Thomas et al., 2018). Atherton and Watson (2015); Atherton et al. (2017) used hyperspectral remote sensing for detection of early blight (*Alternaria solani*) in potato plants prior to visual disease symptoms. In this case only spectral information was used as the authors didn’t use an imaging sensor. For late blight (*Phytophthora infestans*) detection Ray et al. (2011) also used a point spectrum approach without using spatial information. Hu et al. (2016) used hyperspectral imaging to detect late blight disease on potato leaves successfully, with a discrimination of 95% between healthy and diseased leaves.

Although virus diseases have a different mechanism by which they change the plant physiology, virus symptoms can also be measured using optical techniques. The TBV was successfully detected in tulip plants using spatial and spectral information (Polder et al., 2010). Spectral signatures of potato plants infected with PVY, acquired with an hand-held device, were classified using a SVM with an accuracy of 89.8% between infected and non-infected plants (Griffel et al., 2018).

For hyperspectral imaging, the entire system pipeline, consisting of the type of sensor, the mobile platform carrying the vision system, and the decision-making process by data analysis has to be tailored to the specific problem (Kuska and Mahlein, 2018).

From the data analysis perspective, the use of multi-scale datasets of hyperspectral images for plant disease detection or the scale transfer of prediction models is a very challenging, emerging topic (Arens et al., 2016; Roscher et al., 2016; Thomas et al., 2018). Spectral vegetation indices have been shown to be useful for an indirect detection of plant diseases at a canopy level. Mahlein et al. (2013) developed specific spectral disease indices for the detection of diseases in sugar beet plants. Advanced machine learning algorithms were used in several studies. Rumpf et al. (2010) developed a SVM classifier for detection of *Cercospora* leaf spot, leaf rust and powdery mildew on sugar beet leaves.

Deep Convolutional Neural Networks have proven to be a powerful tool for disease detection based on RGB color images (Sladojevic et al., 2016; Fuentes et al., 2017; Pound et al., 2017). With adaptations this technique can also be successfully applied to hyperspectral images (Garcia-Garcia et al., 2017). Chen et al. (2014) proposed a hybrid framework of PCA, deep learning architecture, and logistic regression for classification of hyperspectral remote sensing data.

Of particular interest to the present article is the fully convolutional neural network (FCN) architecture first proposed by Long et al. (2015). This class of neural network provides an elegant means of performing semantic segmentation of images. The core principle underlying this class of neural network is the replacement of the final fully connected classification stage of standard CNNs with a pixel-level segmentation stage which uses convolution and upsampling techniques to replicate the same computation across an entire image in one forward pass. As discussed in Garcia-Garcia et al. (2017), subsequent studies have further elaborated on the method.

A review of hyperspectral image analysis techniques for the detection and classification of the early onset of plant disease and stress is given by Lowe et al. (2017). A central focus of that review is the utilization of hyperspectral imaging in order to find additional information about plant health, and the ability to predict onset of disease.

This paper focuses on the detection of PVY infected potato plants using hyperspectral imaging and deep learning. A novel FCN is used to detect plant diseases based on hyperspectral image data.

## Materials and Methods

### Experimental Field

The experimental field was located in the central polder area of the Netherlands. This farm land was reclaimed from the sea in the 1940’s and is a high-quality clay soil. The field was part of an experimental field of the Dutch General Inspection Service (NAK) near Emmeloord (Netherlands).

**FIGURE 1:**
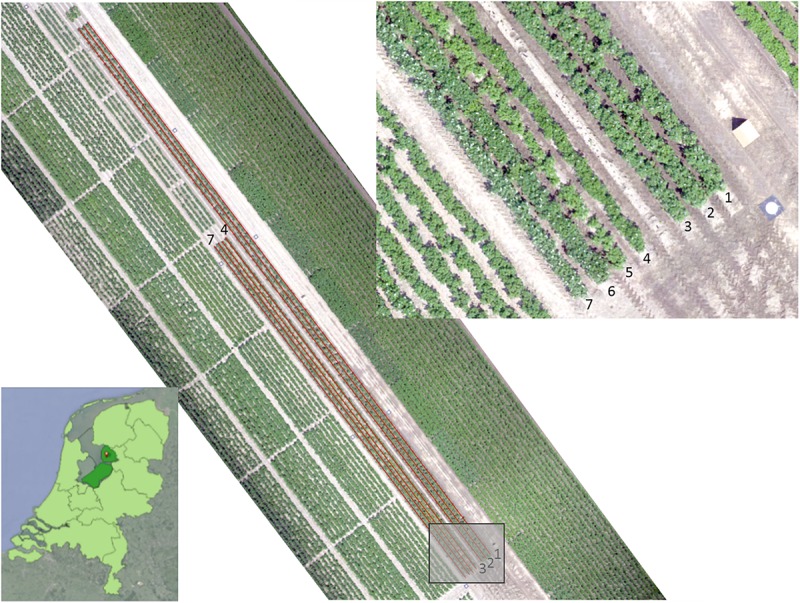
RGB image of the experimental field, acquired with UAV on June 19, 2017. The length of the rows is 110 m for rows 1–3 and 66 m for rows 4–7. The location is near Tollebeek in the Netherlands (lower left).

Figure 1 shows an image of the field layout. Tubers were planted on the 11th of May with an intra-row distance of 33 cm. Table 1 shows information on the varieties and infections used in the different rows. Rows 1–3 contained plants that were infected with bacterial diseases, where rows 4–7 contains plants from 4 different varieties infected with PVY. The PVY infected potato batches were collected by the NAK and selected for a fairly level of infection.

**Table 1:** Description of the test field.

Row	Cultivar	Row length [m]	Number of tubers	Infection
1	Kondor	110	333	Erwinia
2	Kondor	110	333	Erwinia
3	Kondor	110	333	Erwinia
4	Rosa Gold	66	200	PVY
5	Lady Claire	66	200	PVY
6	Vermont	66	200	PVY
7	PCR/11	66	200	PVY

Cultivation practices were applied according to practice. The fertilization basis consisted of the normal amounts of NPK, with an additional dosage of Manganese Nitrate (total: 0,2 kg Mn) and Magnesium Nitrate (total: 1.1 kg Mg). Crop protection to late blight was limited to 13 applications.

Weather conditions during the growing season were quite different. After a cold spell in March (before planting), the month of April was quite normal (cool, limited amount of rain) followed by a long warm and dry period in May and June. By the end of June, the weather changed and the month of July was wet and started cool.

During the course of the experiment, all plants in the field were visually monitored several times by an experienced inspector of the NAK.

As can be seen from Table 1, rows 1, 2, and 3 had mainly bacterial infections, but some natural occasional Y virus infections occurred. These plants as well as the healthy plants in the first 3 rows were used for training the CNN. Plants showing symptoms caused by bacterial pathogens were excluded from the analysis.

Rows 4–7 contained the virus infected plants. Unfortunately, plants in row 5 (Lady Claire) appeared to be 100% symptomatic and plants in row 4 (Rosa Gold) showed more than 95% symptomatic plants. Furthermore, Potato Virus X (PVX) symptoms on row 4 disturbed the manual scores of the crop experts. The appearance of PVX was confirmed by a laboratory assay (ELISA). Therefore row 4 and 5 were excluded from the hyperspectral analysis.

Measurements were done at a weekly interval during the growing season, starting 6 weeks after planting when plants just started to cover each other. Due to the late start of the measurements the symptomatic plants stabilized at 13 for row 6 and 8 for row 7 (Tables 3, 4).

### Hyperspectral Image Acquisition

**FIGURE 2:**
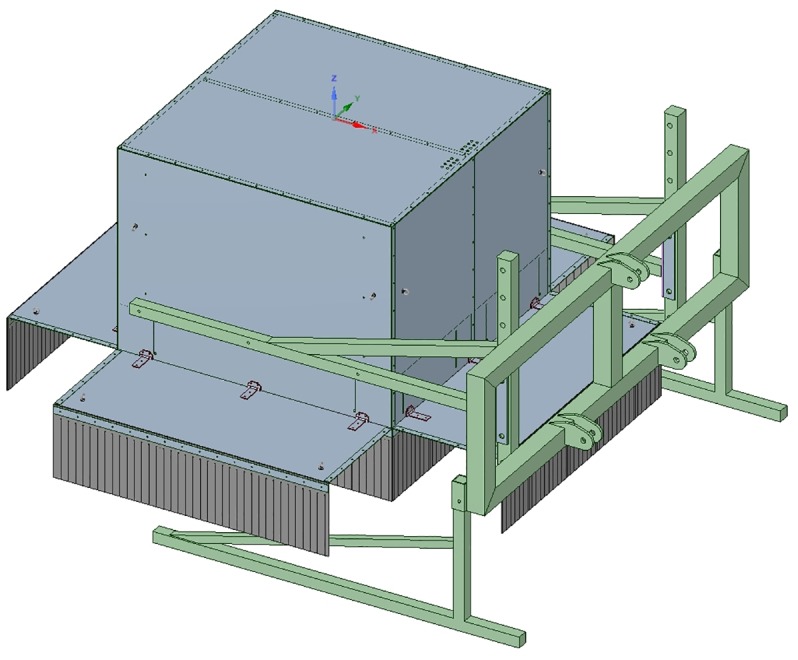
Drawing of the measurement box, consisting of two compartments, the first one for the hyperspectral camera and the second one for an RGB-D imaging system. Ambient light is blocked by two rows of rubber flaps. The box can be mounted on the front of a tractor.

**FIGURE 3:**
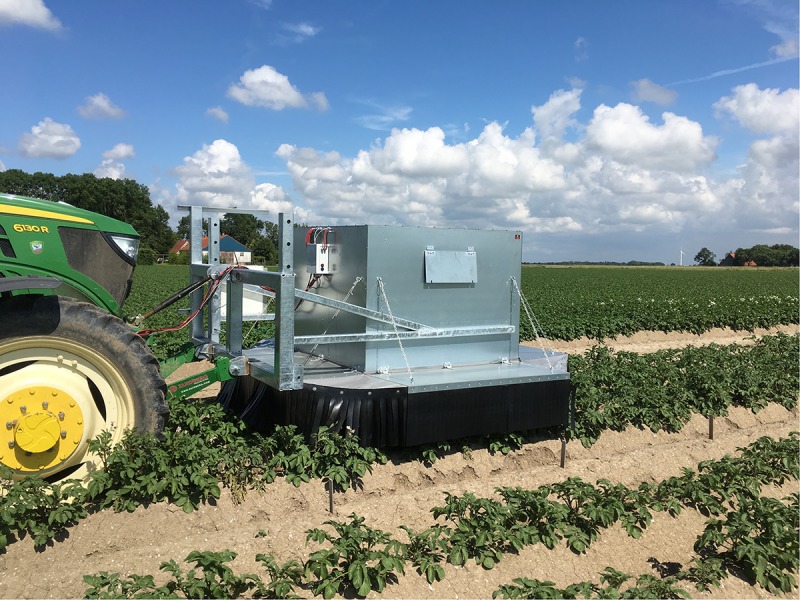
Picture of the system while doing field measurements.

**FIGURE 4:**
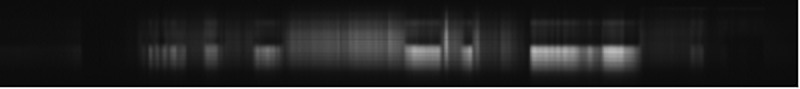
One single hyperspectral line image, horizontally showing the spatial information of one line, vertically showing the spectral reflection between 400 and 1000 nm.

Image acquisition was done in a larger scope where several sensor techniques were explored for disease detection in the field. An imaging box was designed for measuring the potato plants. The box consists of two equally sized compartments (150 cm × 75 cm × 150 cm). The first compartment was equipped with an RGB-Depth camera while the other was equipped with a Specim FX10 hyperspectral line scan camera (wavelength range 400–1000 nm). For this paper we only focussed on the hyperspectral data. An embedded PC (Nexcom NISE3500) was installed to acquire the hyperspectral images. Ambient light was blocked by use of light curtains placed around the measurement box (Figure 2). The measurement box was placed 3.1 m in front of a tractor that drove at a constant speed of 300 m per hour (0.08 m/s) during the measurements. Figure 3 shows the system operating in the test field. The FX10 is a pushbroom hyperspectral line scan sensor. The frame rate was 60 f/s, resulting in an interval of 5 mm in the driving direction of the tractor. The full sensor resolution of the FX10 is 1024 pixels in the spatial by 224 bands spectral. In order to improve light sensitivity and speed, the images were binned by a factor 2 in the spatial direction and a factor 4 in the spectral direction, resulting in line images of 512 pixels × 56 pixels (Figure 4). As the bands at the start and the end of the spectrum were noisy, only the central 35 bands were kept for further processing.

Plants were illuminated by 13 Tungsten Halogen lamps (Osram Decostar 51 PRO, 14 Watt, 10°, Dichroic) placed in a row. This way the plants were evenly illuminated. White and black references were taken at the start of each measurement cycle. The white reference object was a gray (RAL 7005) PVC plate. The black reference was taken with the camera shutter closed. Reflection images were calculated by:

$$R=\frac{I-B}{W-B}$$

where *I* is the raw hyperspectral Image, *B* is the black reference and *W* is the White reference.

The total length of the crop rows were 110 m and 66 m for rows 1–3 and 4–7, respectively, with an interval of 5 mm, a total number of 22,000 and 13,200 line images per row were acquired.

### Geo Referencing

The health status of each potato plant was determined by a crop expert who visually inspected the plants in the experimental field. Plants that showed Y-virus disease symptoms were geometrically stored with a RTK GNSS rover (Hiper Pro, Topcon, Tokyo, Japan). A VRS signal (06-GPS, Sliedrecht, Netherlands) was used to guarantee a 0.02 m accuracy on the position estimate. The crop expert obtained the position of a diseased plant by placing the rover at the center point of the plant. From the center point, we constructed a geometric plant polygon using the intra- (0.33 m) and inter-row (0.75 m) distance of the potato crop. The constructed plant polygons were stored for offline processing.

On each measurement day, we obtained the real-world position of the hyperspectral line images using the RTK-GNSS receiver of the tractor (Viper 4, Raven Europe, Middenmeer, Netherlands). The GGA message of the GNSS receiver (NMEA-0183), containing the WGS-84 coordinates and the GNSS precision status, were passed to the embedded PC at a frequency of 10 Hz and stored for offline processing.

**FIGURE 5:**
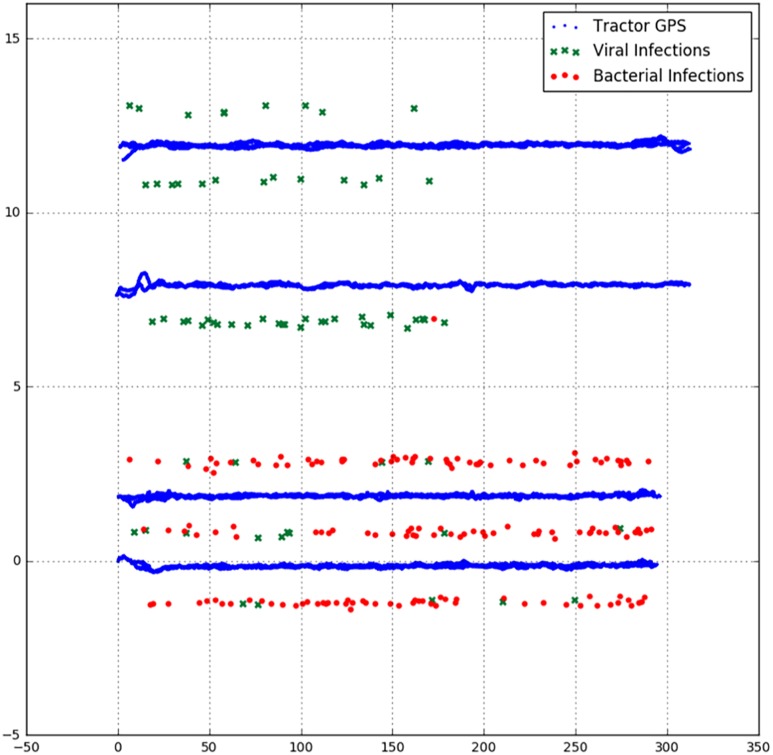
The GNSS positions of the RTK rover, labeled as viral or bacterial infection and the GNSS position of the tractor driving back and forth over the rows. The units on the axes are scaled to half the inter row distance and uniform in both directions.

We processed the data in such a way that only the hyperspectral line images were assessed where a RTK-fix signal was guaranteed. In this way the exact position of all hyperspectral images could be determined with a precision of 0.02 m. First, the obtained GNSS coordinates were translated to 3.1 m in front of the tractor to correspond to the real-world position of the hyperspectral images. Furthermore, the WGS-84 coordinates of the images were projected to the planar coordinate system of the Netherlands (RD-new). These planar coordinates were rotated and scaled such that the crop rows were parallel in the X-direction. For each hyperspectral line, we determined the health status by checking the intersection between the line and the geometric plant polygon. Lines that overlapped more than one polygon were left out of consideration. Figure 5 shows a plot of the GNSS positions of the RTK rover, labeled as viral or bacterial infection and the GNSS position of the tractor driving back and forth over the rows.

### Deep Learning

The network used was a fully convolutional neural network (FCN), but had a non-standard decoder (final) portion. Usually with FCNs the output is a two-dimensional segmentation. Here, we outputted a one-dimensional segmentation (it was also a lower resolution 1D strip).

Because the training data is imbalanced (a lot more healthy cases than diseased cases), the data was resampled in order to emphasize diseased examples. As deep learning needs huge numbers of training data, the available data was enriched by data augmentation techniques, such as random mirroring and rotation, as well as randomized changes in image brightness.

The neural network architecture employed was an adaptation of the FCN family of approaches. FCNs are particularly well suited to performing semantic segmentation of two-dimensional images, where one wants to assign a class (such as “human” or “dog”) to each pixel of the image. FCNs employ only a combination of convolutions, pooling, unpooling and per-pixel operations (such as ReLU non-linear activation functions). This allows FCNs to produce a segmentation for the entire image in one forward pass, replicating the same computation for each region of the image.

Instead of a two-dimensional segmentation, we employed a similar approach to predict a one-dimensional stream of labels, with the goal of assigning a label to each hyperspectral line image.

**FIGURE 6:**
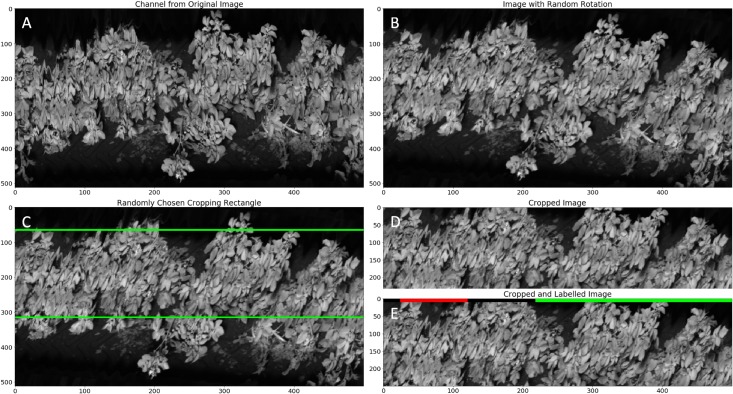
Illustration of the procedure for generating a training image. The figure shows the 722 nm wavelength band. The original image is 500 × 512 **(A)**. This image is then rotated by a random angle uniformly distributed between –10° to 10° **(B)**. Subsequently a randomly positioned rectangle with dimensions 500 × 250 is cropped from the rotated image **(C)** to produce the training image **(D)**. The procedure for test images is the same, except there is no rotation step and the cropping rectangle is always vertically centered. Ground truth labeling is done on line bases (green – healthy, red – diseased, black – unknown) **(E)**.

Each of the input images is constructed from 500 consecutive line scan images, representing a subset of a row captured by the tractor. From each line scan image of 512 pixels, 250 pixels are retained. During testing, these are the central 250 pixels, but the interval is randomly chosen during training as a form of data enrichment. The process of generating a training or test image is illustrated in Figure 6. Figure 6E illustrates the annotation of the input images based on the health status of each potato plant as determined by the crop expert and stored with the (RTK-GNSS) rover. Ground truth is indicated in the top band, with green and red labels indicating healthy or diseased plants, respectively. Black labels indicate regions excluded from testing due to labeling uncertainty. Input images have a resolution of 500 × 250. The number of feature maps at the input equals the number of hyperspectral bands kept (35).

**FIGURE 7:**
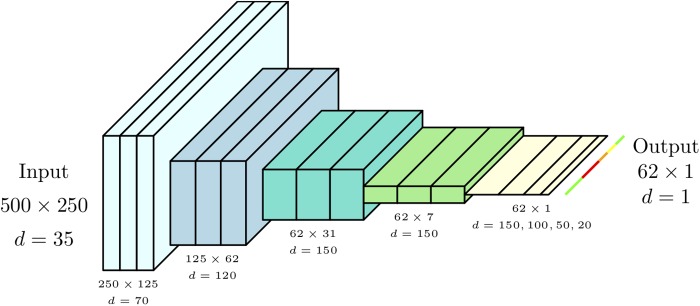
Neural Network Architecture consisting of 17 stages, each of which contains a convolutional layer followed by a number of optional layers including max pooling.

The network architecture is described in Table 2 as a series of 17 stages, each of which contains a convolutional layer followed by a number of optional layers. These optional layers include max pooling, a per-element non-linearity (either Leaky ReLU activation functions with negative gradient of 0.01, or sigmoidal), batch normalization and dropout (Figure 7). A dropout probability of 0.5 was used. A SpatialDropout layer (Tompson et al., 2015) was used toward the start of the convolutional portion of the network (within stage 1). Standard dropout layers are employed in the fully connected portion of the network (stages 14 through 16).

**Table 2:** Neural Network Architecture consisting of 17 stages, each of which contains a convolutional layer followed by a number of optional layers including max pooling.

Stage number	Kernel size	Stride	Maxpool	Non-linearity	Batch normalization	Dropout (*p* = 0.5)	Resulting feature maps	Resulting resolution
**Input**							35	500 × 250
**1**	7 × 7	2 × 2		Leaky ReLU	Yes	Spatial	70	250 × 125
**2**	3 × 3	1 × 1		Leaky ReLU	Yes	No	70	250 × 125
**3**	3 × 3	1 × 1		Leaky ReLU	Yes	No	70	250 × 125
**4**	3 × 3	1 × 1	2 × 2	Leaky ReLU	Yes	No	120	125 × 62
**5**	3 × 3	1 × 1		Leaky ReLU	Yes	No	120	125 × 62
**6**	3 × 3	1 × 1		Leaky ReLU	Yes	No	120	125 × 62
**7**	3 × 3	1 × 1	2 × 2	Leaky ReLU	Yes	No	150	62 × 31
**8**	3 × 3	1 × 1		Leaky ReLU	Yes	No	150	62 × 31
**9**	3 × 3	1 × 1		Leaky ReLU	Yes	No	150	62 × 31
**10**	3 × 3	1 × 1	1 × 4	Leaky ReLU	Yes	No	150	62 × 7
**11**	3 × 3	1 × 1		Leaky ReLU	Yes	No	150	62 × 7
**12**	3 × 3	1 × 1		Leaky ReLU	Yes	No	150	62 × 7
**13**	3 × 3	1 × 1	1 × 4	Leaky ReLU	Yes	No	150	62 × 1
**14**	1 × 1	1 × 1		Leaky ReLU	No	Yes	100	62 × 1
**15**	1 × 1	1 × 1		Leaky ReLU	No	Yes	50	62 × 1
**16**	1 × 1	1 × 1		Leaky ReLU	No	Yes	20	62 × 1
**17**	1 × 1	1 × 1		Sigmoid	No	No	1	62 × 1

*SpatialDropout was employed in stage 1.*

Stages 1 through 7 are typical of two-dimensional FCNs. Here we perform a series of convolutions which act as successive feature extraction stages. Interspersed max pooling stages lower the feature map resolution, allowing the convolutions to extract features based on larger regions of image context. The feature map spatial dimensionality reduces in a symmetrical fashion, leading to more enriched and discriminating features.

Stages 8 through 13 are similar to earlier stages, except here the architecture departs from two-dimensional FCNs in that we now restrict pooling to the dimension parallel with individual line images. There were two sections (stages 8–10 and 11–13) containing 3 convolutions each, with both sections ending with an 1 × 4 max pooling. This had the effect of asymmetrically shrinking the feature map resolution from 62 × 31 to 62 × 7, and then finally 62 × 1. We refer to these two stages as “combiners,” as they were meant to combine evidence across a hyperspectral line (and its close neighbors) until this spatial dimension has collapsed to a length of 1. Additionally, the two sections shared the same parameters (as indicated in Table 2), which was motivated by both these sections performing the operation of pooling evidence across local regions into larger regions.

Stages 14 through 17 performed the final per-label decision through a series of convolutions with 1 × 1 kernels. Due to the kernel dimensions, these were effectively fully connected stages performed in parallel, and independently, on each output point. The final layer has a sigmoidal non-linearity, as the output should be a probability of disease.

The output layer produced 62 labels, while there were 500 pixels along the corresponding axis in the input image. Typically, a two-dimensional FCN would have included a series of trainable convolutions and unpooling stages to produce a segmentation at the original input resolution. However, in our application, we were concerned with labeling entire plants as healthy or diseased. Since we expect each plant to occupy multiple sequential labels even at the lower resolution of the output, we forgo a trainable unpooling network in favor of a simple nearest neighbor upscaling to 500 pixels to match the input image.

Ground truth labeling made use of GNSS coordinates of diseased plants (as determined by crop experts). Training and testing the neural network require labeling of each hyperspectral line image (either as diseased, healthy or excluded). To convert the GNSS coordinates to ground truth labeling, the following procedure was employed. Initially, all line images were labeled as healthy. Then, for each diseased plant, the line image with GNSS tag closest to the diseased plant was located, which acted as a center point for subsequent labeling. The 150 line images before and after this center line were marked as excluded (if not already marked as diseased). Then, if the infection was specifically of a viral nature, the 50 line images before and after the center point were marked as diseased.

Because there was non-uniformity in the dimensions and growth patterns of each individual plant, this labeling procedure ensured that we labeled only regions where there was a high degree of certainty regarding the health of associated plants.

Figure 6 shows an example of such a labeling where a single plant with a viral infection is located toward the left-hand side of the image. The core diseased labels and excluded regions can be seen surrounding this plant, with regions marked as healthy extending beyond the excluded region.

During training and testing, predictions were produced for all hyperspectral line images in the input regardless of their labeling. However, only lines that had ground truth labeling as either healthy or diseased contributed to error measures used during training and evaluation of the system, whereas excluded lines did not contribute.

The model was trained on rows 2 and 3, with row 1 used as a validation set during the training process. After training was completed, the resulting neural network was independently tested on rows 6 and 7.

The model was trained and tested on a system containing an Intel Xeon E5-1650 CPU with a 3.5 GHz clock speed and 16 Gb of RAM. The system also housed an Nvidia GTX 1080 Ti GPU with 11 Gb of video memory. All data preprocessing, Deep Learning training and validation was done using the PyTorch deep learning framework (Paszke et al., 2017). At test time, a forward pass through the neural network took a total of 8.4 ms, of which 4.0 and 1.1 ms were the times taken to move data to and from the GPU, respectively. This time is negligible compared to the time taken to acquire the images.

## Results

**FIGURE 8:**
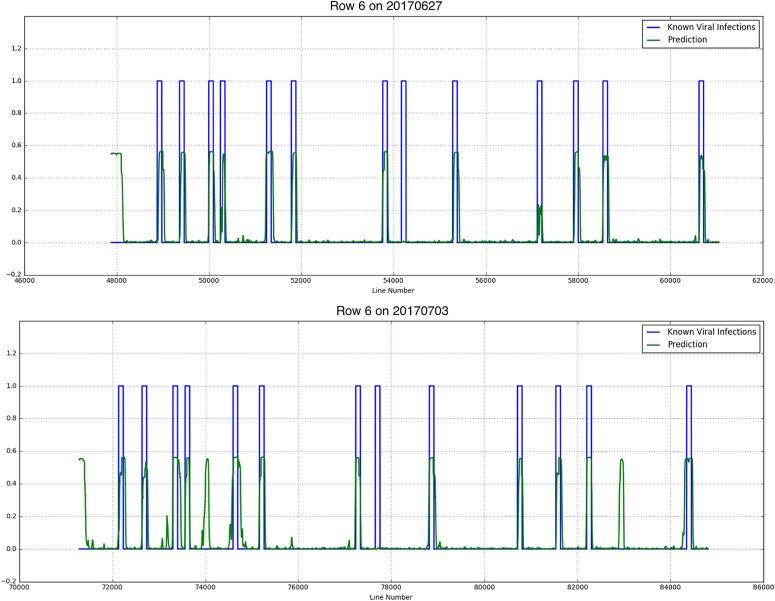
Known PVY infection and CNN predicted infection for row 6 on 2017/06/27 and 2017/07/03.

Results showed that although there were not much Y Virus infected plants in the training set, the network performed well in predicting the infected plants in row 6 and 7. Figure 8, 9 show the results for the different measurement dates for row 6 and 7, respectively. In these figures the *x*-axis shows the line number in the hyperspectral image of the total row. The *y*-axis shows the output of the network as a probability. An average of network predictions was calculated over all line images associated with a particular plant, and a probability threshold of 0.15 on this average was used to distinguish the diseased plants for prediction (Figure 10).

**FIGURE 9:**
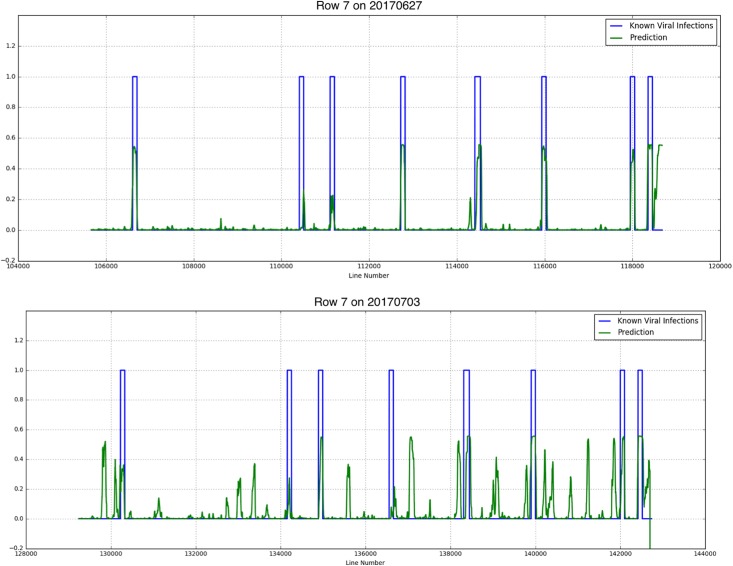
Known PVY infection and CNN predicted infection for row 7 on 2017/06/27 and 2017/07/03.

**FIGURE 10:**
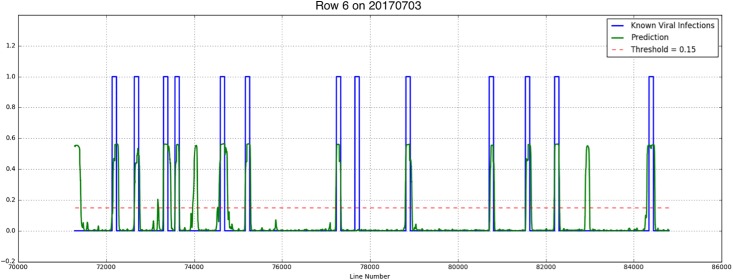
A threshold of 0.15 was applied to the average predictions for a given plant in order to translate the probability output of the CNN into a decision.

In Table 3 the confusion matrices after classification are given for the different rows and measurement dates. From the confusion matrices the precision (also called positive predictive value) and recall (also known as sensitivity) were calculated. Precision is a measure of the fraction of relevant instances, in this case the diseased plants among the retrieved instances, while recall (also known as sensitivity) is the fraction of relevant instances that have been retrieved over the total amount of relevant instances (Olson and Delen, 2008). Precision and recall are then defined as:

**Table 3:** Confusion matrices of row 6 **(A,B)** and 7 **(C,D)**, measured on 2017/06/27 **(A,C)** and 2017/07/03 **(B,D)**.

	Predicted PVY	Predicted healthy
**A**
Known PVY	12 (92.3%)	1 (7.7%)
Known healthy	11 (6.4%)	160 (93.6%)
**Recall: 0.92, Precision: 0.52.**		
**B**
Known PVY	12 (92.3%)	1 (7.7%)
Known healthy	18 (10.5%)	153 (89.5%)
**Recall: 0.92, Precision: 0.4.**		
**C**
Known PVY	7 (87.5%)	1 (12.5%)
Known healthy	6 (3.4%)	173 (96.6%)
**Recall: 0.88, Precision: 0.54.**		
**D**
Known PVY	6 (75%)	2 (25%)
Known healthy	20 (11.1%)	159 (88.9%)
**Recall: 0.75, Precision: 0.23.**		

$$\begin{array}{c} Precision=\frac{tp}{tp+fp}\\ Recall=\frac{tp}{tp+fn} \end{array}$$

where *tp* is the true positive fraction, *fp* is the false positive fraction and *fn* is the false negative fraction. For row 6 the precision is 0.52 and 0.4 for the first and second measurement week, respectively, and the recall is 0.92 for both weeks. For row 7 the precision measures are 0.54 and 0.23, respectively, the recall is 0.88 and 0.75, respectively.

**FIGURE 11:**
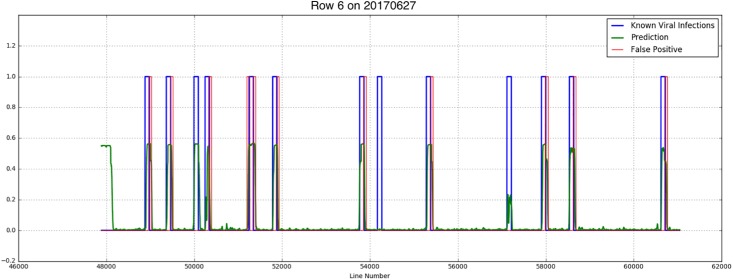
False positives are closely connected to true positives.

Investigation into the position of the FPs shows that most of them were connected to TPs (Figure 11). When the FPs connected to TPs were ignored, the confusion matrices are much better (Table 4). The recall measures stay the same, as this correction does not affect the FNs. The precision measures improve to 0.92 and 0.8 for row 6 and 0.78 and 0.3 for row 7, respectively.

**Table 4:** Confusion matrices of row 6 **(A,B)** and 7 **(C,D)**, measured on 2017/06/27 **(A,C)** and 2017/07/03 **(B,D)**, after correction for neighboring plants.

	Predicted PVY	Predicted healthy
**A**
Known PVY	12 (92.3%)	1 (7.7%)
Known healthy	1 (0.6%)	170 (99.4%)
**Recall: 0.92, Precision: 0.92.**		
**B**
Known PVY	12 (92.3%)	1 (7.7%)
Known healthy	3 (1.8%)	168 (98.2%)
**Recall: 0.92, Precision: 0.8.**		
**C**
Known PVY	7 (87.5%)	1 (12.5%)
Known healthy	2 (1.1%)	177 (98.9%)
**Recall: 0.88, Precision: 0.78.**		
**D**
Known PVY	6 (75%)	2 (25%)
Known healthy	14 (7.8%)	165 (92.2%)
**Recall: 0.75, Precision: 0.3.**		

## Discussion

During viral infections plants react with changes in the respiration, photosynthesis, sugar metabolism and phytohormone activity. This results in (pathological) changes that are observed at a microscale in the form of an increased number of mitochondria, a degeneration of chloroplasts and a thickening of the cell wall. At a macroscale infected plants show growth reduction, wilting, necrosis, chlorosis and a strong increase of the autofluorescence (Hinrichs-Berger et al., 1999; Kogovsek et al., 2010). Current practice is to manually score the plants in the field using a predefined protocol (NAK) in which 40% of the plants are scored. The accuracy of an experienced judge is 93%. Furthermore, certified personnel need extensive field experience before they can become familiar with the symptoms induced by various pathogens in many potato varieties under differing environmental conditions (Shepard and Claflin, 1975).

In this research we have proven that disease symptoms can be detected with machine vision techniques using hyperspectral cameras with a precision which is almost equal to the accuracy of an experienced crop expert and the possibility of scanning the whole field compared to 40% as defined by the NAK protocol.

For classification an FCN was used. Although these networks are mainly applied to color RGB images, in this study we demonstrated that a modified Fully CNN can be employed on hyperspectral images for the task to detect plant diseases. There are currently very few complete studies applying deep learning to hyperspectral data, though this is an active research area. Several challenges need to be addressed in order to use hyperspectral data for deep learning, including the size of the data and the noisiness of specific wavelength bands (Lowe et al., 2017).

Typically, CNNs are either used for classifying entire images (label-per-image) or providing two-dimensional segmentations (label-per-pixel). Label-per-image approaches generally require vast amounts of training data to generalize properly, input image/label combinations easily lead to overfitting. By contrast, label-per-pixel approaches tend to need smaller input image datasets, as each labeled pixel becomes, in effect, a training case. However, obtaining pixel-level labeling can be difficult, especially if one is searching for subtle image patterns that a human cannot discern in input images.

Instead of either of these extremes, we showed that a “weak” one-dimensional label sequence can be used in combination with a modified FCN architecture for disease detection. This approach has the advantage of increasing the effective number of labels (label-per-line) available in the training set, thus lowering the risk of overfitting. Simultaneously, this approach substantially lowers the burden of labeling datasets. Instead of being required to provide pixel-level annotations, one can use GNSS locations of diseased individuals to generate ground truth on the line-level, which is a substantially simpler process. We also showed that the system is robust with respect to uncertainty in the exact boundaries between neighboring plants.

Results showed that although the training data is limited, the prediction of PVY is good. The percentage of detected infected plants, expressed in the recall values, is slightly lower (75–92%) than the accuracy of the crop expert (93%). Due to the low percentage of diseased plants, the precision values are worse, with a range from 0.23 to 0.54 (Table 3). Note that for the crop expert the number of FPs as expressed in the precision measures is unknown. A large amount of the FPs are due to inaccuracy of the positioning system, either on the tractor, or on the RTK rover positioning device when doing ground truth measurements. After correction the precision measures almost doubled for all row/week combinations.

The experiment was setup for detection of both bacterial and virus diseases in seed potatoes. This paper focuses on the detection of PVY virus infected potato plants. Detection of bacterial diseased plants will be reported separately.

The plants were already large and overlapping when the first imaging experiments started. It is expected that when plants are smaller and do not overlap, the accuracy of position of the plant polygons can be greatly improved, which may result in better performance.

It is important to note that the independent test set consisted of plants from other cultivars than those in the training and validation sets. Generally, one does not expect models to generalize well to new varieties as different growth patterns and surface characteristics may disturb regularities observed by the model specific to the training set cultivar. Despite this added difficulty, the system obtained accuracies with precision larger than 0.75 for 3 out of 4 row/week combinations in the test set. This is a strong indication that the system has found real underlying regularities due to the disease state. We do note that the system performance for the last measurement week of row 7 is relatively poor, indicating the need for a follow-up study to determine the reason for this discrepancy. A potential reason might be that uninfected plants of this variety also start deteriorating due to the heat or plant growth stage.

## Data Availability

The datasets generated for this study are available on request to the corresponding author.

## Author Contributions

GP, PB, JvdW, and JK: contributions to conception and design of this study. GP, PB, HdV, JvdW, and JK: participation in drafting and revising of the manuscript. GP and PB: experiments and data acquisition. JvdW: pathogen inoculation. PB: geo referencing the data. GP and HdV: hyperspectral data analysis.

## Conflict of Interest Statement

The authors declare that the research was conducted in the absence of any commercial or financial relationships that could be construed as a potential conflict of interest.

## Abbreviations

CNN: convolutional neural network
CPU: central processing unit
FCN: fully convolutional network
FN: false negatives
FP: false positives
GGA: message containing time, position, and fix related data
GNSS: global navigation satellite system
GPS: global positioning system
GPU: graphical processing unit
NAK: Nederlandse Algemene Keuringsdienst (Dutch General Inspection Service)
NMEA: The National Marine Electronics Association, standard for GNSS position information
NPK: nitrogen, phosphorus and potassium
PCA: principal component analysis
PVC: polyvinyl chloride
PVY: potato virus Y
RAL: color standard
RAM: random access memory
RD-New: Local coordinate reference systems of the Netherlands
ReLU: rectified linear unit
RGB: red, green, blue
RGB-D: red, green, blue and depth
RTK: real-time kinematic
SVM: support vector machine
TBV: tulip breaking virus
TP: true positives
UAV: unmanned aerial vehicle
VRS: virtual reference station
WGS-84 -: World Geodetic System, latest revision, 1984
